# Cavitation and durable remission in a PD-L1–positive, TP53-mutant SMARCA4-deficient lung tumor following immunochemotherapy and radiotherapy: A case report

**DOI:** 10.1016/j.rmcr.2026.102390

**Published:** 2026-02-26

**Authors:** Kelan Deng, Guixia Liu, Yinping Li, Peiling Bao, Yue Zhang, Zhibin Xie

**Affiliations:** Department of Respiratory and Critical Care Medicine, Xiaogan Hospital Affiliated to Wuhan University of Science and Technology, Xiaogan, Hubei, 432000, China

**Keywords:** SMARCA4-deficient tumor, PD-L1, TP53 mutation, Cavitation, Immunochemotherapy, Radiotherapy

## Abstract

SMARCA4-deficient undifferentiated tumors (SMARCA4-UTs) are rare, aggressive thoracic malignancies with dismal prognosis. Most cases are refractory to conventional therapy, a significant number of patients show no response to one or more chemotherapy regimens, exhibiting an overall survival (OS) of less than six months. We report a case of a 52-year-old male heavy smoker with a right lower lobe SMARCA4-UTs harboring TP53 mutation and PD-L1 expression (tumor proportion score [TPS] 30%, combined positive score [CPS] 30%). The tumor showed loss of SMARCA4 (also known as BRG1), high Ki-67 (∼50%), and rapid growth. After six cycles of tislelizumab + paclitaxel/cisplatin, the lesion demonstrated partial remission and progressive cavitation on CT imaging. Consolidative radiotherapy (60 Gy/30 fractions) further reduced the tumor burden. The survival period of this patient was 17 months long. This case highlights that PD-L1 expression and radiologic cavitation may serve as potential efficacy biomarkers in SMARCA4-UTs, even in tumors with TP53 mutations and a high proliferative index. Combined immunotherapy with chemotherapy and radiotherapy may confer durable disease control in this aggressive lung cancer subtype.

## Highlights

• In SMARCA4-UT, PD-L1 expression & cavitation may predict immunochemotherapy efficacy, even with TP53 mutation. Combo therapy offers durable control.

## 1 Introduction

SMARCA4-UTs are a rare and aggressive malignant tumors first reported by Le Loarer et al., in 2015 [1]. In 2021, the World Health Organization (WHO) recognized it as a distinct disease entity and established unified diagnostic criteria in the fifth edition of the International Classification of Diseases. Clinically, it is primarily differentiated from traditional SMARCA4-deficient non-small cell lung cancer (SMARCA4-dNSCLC) through pathological features [2]. This tumor predominantly affects middle-aged male smokers, with nonspecific clinical and imaging features that pose diagnostic challenges. Definitive diagnosis requires pathological and immunohistochemical analysis. Histopathologically, it comprises epithelioid and rhabdomyoid tumor cells exhibiting mixed sarcoma and carcinoma characteristics. Immunohistochemistry detects the loss of BRG1 expression in SMARCA4-deficient cells, indicating disrupted chromatin remodeling pathways that lead to abnormal cell differentiation, uncontrolled proliferation, and tumor progression [3,4].

Owing to the rarity, there is no unified standard treatment protocol recommended by current guidelines. Most patients with this tumor have poor treatment responses and the median survival period is less than half a year.

This report describes a case of SMARCA4-UTs with PD-L1 expression and TP53 mutation. The patient received an immunotherapy combined with chemotherapy and radiotherapy, achieving durable disease control while developing radiological cavitary sign—a treatment-related manifestation that, though uncommon, holds clinical significance.

## 2 Case presentation

**Fig. 1:**
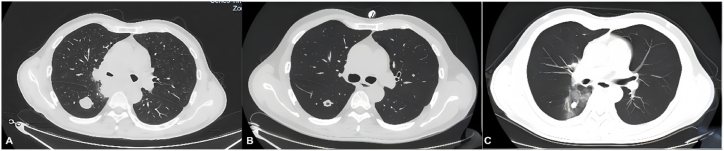
Serial contrast-enhanced CT scans. (A) May 2024: 2.14 × 2.45 cm lobulated mass in right lower lung with mediastinal/hilar lymphadenopathy. (B) September 2024: Tumor reduced to 1.6 × 1.2 cm with cavitation after immunochemotherapy. (C) February 2025: Further reduction to 1.4 × 1.0 cm, cavitation persisted, no new metastases.

**Fig. 2:**
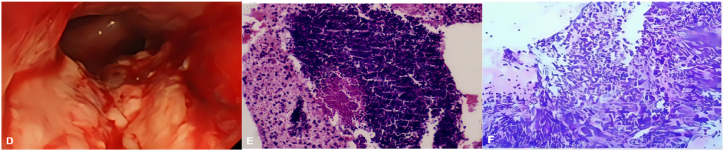
Histology and immunohistochemistry. (D) Bronchoscopy: neoplastic growth partially obstructing right main bronchus. (E) PD-L1 expression (HE, × 40): tumor cells with TPS 30%, CPS 30%. (F) EBUS-TBNA pathology (IHC, × 40): BRG1 loss, Ki-67 ∼50%, p53 mutant phenotype.

A 52-year-old male patient was admitted in May 2024 due to a one-month history of a persistent right lower lung space-occupying lesion and a week-long history of hemoptysis. The patient reported discovery of this lesion during a physical examination one month ago (no imaging or medical documentation available) and did not seek treatment at that time. Hemoptysis began one week ago, initially presenting as intermittent streaks of fresh, red blood in the sputum without specific intervention. The blood volume gradually increased but remained below 5 mL per episode. The patient had a 20-year smoking history (40 cigarettes per day) and had quit smoking five months prior to admission. On admission, chest auscultation revealed coarse bilateral breath sounds without rales, with no other physical abnormalities. After admission, pulmonary tumor markers, including carcinoembryonic antigen (CEA), neuron-specific enolase (NSE), cytokeratin 19 fragment (CYFRA21-1) and squamous cell carcinoma antigen (SCCA), were all within normal limits/negative. Initial CT imaging (Fig. 1A) showed a lobulated hyperdense lesion (2.14 × 2.45 cm, CT value of approximately 15HU) in the right lower lobe, accompanied by mediastinal and right hilar lymphadenopathy, with airway narrowing and signs of obstructive pneumonia. No enlargement was observed in cervical, axillary, or inguinal superficial lymph nodes, and no metastatic lesions were detected on cranial or abdominal CT. Endobronchial ultrasound-guided transbronchial needle aspiration (EBUS-TBNA)revealed enlargement of group 7, 10R, and 11Ri lymph nodes, and histological examination confirmed to be undifferentiated carcinoma with BRG1 expression loss. Molecular analysis identified TP53 mutations (45.72%) and PD-L1 positivity (TPS 30%, CPS 30%) (see Fig. 2).

Immunohistochemistry results were as listed in Table 1.

**Table 1:** Immunohistochemistry results.

Category	Marker	Result
Epithelial	CK7, TTF-1, Napsin-A, PCK	Negative
Squamous	CK5/6, p40	Negative
Mesenchymal	Vimentin	Positive
SMARCA4/INI1	BRG1 (SMARCA4)	Negative
	INI1	Positive
Proliferation/Tumor suppressor	Ki-67	50%
	p53	Mutant phenotype

Combined with systemic evaluation and pathological findings, the diagnosis was SMARCA4-deficient undifferentiated carcinoma, stage cT1bN2bM0 IIIA, with a PS score of 0. From May to September 2024, the patient received six cycles of tislelizumab (200 mg) combined with paclitaxel (400 mg) and cisplatin (40 mg, administered on D1-3). Follow-up enhanced CT scans (Fig. 1B) revealed: (1) Tumor shrinkage to 1.6 × 1.2 cm; (2) Cavity formation in the right lower lung lesion; (3) Significant reduction in the size of mediastinal and hilar lymph nodes. The treatment was assessed as a partial response (PR) after six cycles. In October 2024, radiotherapy was administered with the following dosage regimen: (1) Gross Tumor Volume—primary (GTVp): 60 Gy/30 fractions; (2) Metastatic lymph nodes (GTVn): 60 Gy/30 fractions; (3) Lymphatic drainage area: 50 Gy/25 fractions, which further reduced the tumor burden. The patient later resumed tislelizumab maintenance therapy at a local hospital. A follow-up chest CT scan (Fig. 1C) in February 2025 showed residual nodules measuring 1.4 × 1.0 cm with persistent cavities and ground-glass opacities, but no evidence of new metastasis. The patient continued tislelizumab maintenance therapy until September 2025 and owing to ultimately succumbed to severe pneumonia in October 2025.

## 3 Discussion

SMARCA4-UTs are rare, aggressive malignant tumors characterized by undifferentiated or rhabdomyoid histology, predominantly affecting the mediastinum. Their gene expression profile overlaps with that of malignant rhabdomyosarcoma [5]. In the 2021 WHO classification system, these tumors have been reclassified as epithelial tumors, owing to their shared genetic background with smoking-associated lung cancer [3]. Primarily occurring in young to middle-aged adults, SMARCA4-UTs can develop into large masses compressing mediastinal and peripulmonary tissues. Current treatment options remain limited, with no standardized therapeutic protocol established. Most patients die within six months owing to high early recurrence rates, poor response to radiotherapy, chemotherapy resistance, and a lack of effective targeted therapies [6]. Studies suggest that combining chemotherapy with immunotherapy may improve progression-free survival (PFS). Immunotherapy could also serve as an effective salvage treatment following chemotherapy failure [7].

Physiopathologically, SMARCA4-UTs exhibit SMARCA4 deficiency, often accompanied by mutations in TP53, STK11, KEAP1, and KRAS, along with a high tumor mutation burden (TMB) [8]. The SMARCA4 gene, located at 19p13.2, encodes a subunit that functions as the core ATPase of the BAF (BRG1/BRM-associated factor) chromatin remodeling complex. This subunit is the catalytic component of the SWI/SNF complex and functions as a critical epigenetic tumor suppressor involved in transcriptional regulation, cell-cycle control, and DNA damage repair. Its loss leads to global chromatin dysregulation, acquisition of stem-like features, profound genomic instability, and highly aggressive tumor behavior. From a pathophysiological perspective, SMARCA4 deficiency impairs homologous recombination and double-strand break repair, promoting a high mutational burden and enhanced tumor immunogenicity — factors that contribute to the tumor's aggressiveness despite a poor baseline prognosis [9].

Moreover, SMARCA4's influence on tumor progression involves the tumor microenvironment (TME). Specifically, M2 macrophage-derived exosomes (MDEs) can downregulate BRG1 and transfer oncomiRs, such as miR-21-5p and miR-155-5p, to cancer cells, thereby enhancing their migratory and invasive capabilities. This deficiency also leads to the loss of CD44 expression — a transmembrane glycoprotein essential for the growth and metastasis of many malignancies [7].

In the present case, the concomitant SMARCA4 and TP53 alterations likely synergized to enhance both radiosensitivity and chemosensitivity by abrogating critical chromatin-based DNA repair pathways and cell-cycle checkpoints. Furthermore, SMARCA4 loss has been linked to increased PD-L1 expression and interferon signaling activation, which provides a mechanistic basis for the favorable and durable response to immuno-chemoradiotherapy observed here. This case highlights the dual role of SMARCA4 deficiency: it serves as a marker of aggressive tumor biology while simultaneously emerging as a potential predictor of treatment vulnerability [10].

Tumor cells subjected to cytotoxic chemotherapy (e.g., paclitaxel or platinum-based agents) undergo direct apoptosis or necrosis. Rapid apoptosis induces central necrosis and cavity formation [11]. Immunotherapy, such as anti-PD-1/PD-L1 immune checkpoint inhibitors, activates the patient's immune system to mount a robust anti-tumor immune response against tumor cells, leading to necrosis or lysis and subsequent vacuole formation. The presence of vacuoles is widely recognized as a clinically significant imaging marker, with patients exhibiting vacuole signs demonstrating better tumor control and improved long-term survival rates [12]. In this patient, tumor expression of PD-L1 (TPS/CPS 30%) was associated with immune checkpoint inhibition responsiveness, supporting PD-L1 as a predictive biomarker for SMARCA4-UTs efficacy. Despite the presence of TP53 mutations and a high Ki-67 index (50%)—two indicators typically associated with poor prognosis—the patient achieved durable remission, demonstrating that immunotherapy may overcome unfavorable genomic features. Vacuolization observed during immunotherapy is relatively rare in lung cancer but may reflect immune-mediated tumor necrosis. Vacuolization could serve as a radiological marker for SMARCA4-UTs efficacy.

In conclusion, this case highlights the potential of multimodal therapy (immune checkpoint inhibitors combined with chemotherapy and radiotherapy) to provide meaningful control for this aggressive tumor subtype.

## 4 Conclusion

PD-L1 positivity and radiologic cavitation may indicate the efficacy of immune checkpoint inhibition in SMARCA4-deficient tumors. Combined immunotherapy with chemotherapy and radiotherapy can provide durable control, even in cases with TP53 mutation and a high proliferation index.

## CRediT authorship contribution statement

**Kelan Deng:** Writing – original draft. **Guixia Liu:** Writing – review & editing, Formal analysis. **Yinping Li:** Formal analysis. **Peiling Bao:** Formal analysis. **Yue Zhang:** Conceptualization. **Zhibin Xie:** Writing – review & editing, Resources.

## Patient consent

Written informed consent was obtained from the patient for publication of this case and accompanying images.

## Funding

No funding was received for this work.

## Declaration of competing interest

The authors declare that they have no known competing financial interests or personal relationships that could have appeared to influence the work reported in this paper.
